# Unique dermal bacterial signature differentiates atopic dermatitis skin from healthy

**DOI:** 10.1128/msphere.00156-25

**Published:** 2025-05-09

**Authors:** Lene Bay, Christopher James Barnes, Blaine Gabriel Fritz, Nanna Ravnborg, Iben Frier Ruge, Anne-Sofie Halling-Sønderby, Sif Ravn Søeborg, Kathrine Hald Langhoff, Claus Lex, Anders Johannes Hansen, Jacob P. Thyssen, Thomas Bjarnsholt

**Affiliations:** 1 Department of Immunology and Microbiology, Costerton Biofilm Center, Faculty of Health and Medical Sciences, University of Copenhagen 4321, Copenhagen, Denmark; 2 Centre for GeoGenetics, Globe Institute, University of Copenhagen 4321, Copenhagen, Denmark; 3 Department of Dermatology, Gentofte Hospital, Copenhagen, Denmark; 4 Department of Clinical Microbiology, Rigshospitalet, Copenhagen, Denmark; University of Michigan-Ann Arbor, Ann Arbor, Michigan, USA

**Keywords:** atopic dermatitis, cutaneous community, dermal microbiota, microbial functionality, skin microbiota, core microbiome

## Abstract

Gaining a deeper understanding of the variation in skin microbiota across habitats and layers provides critical insights into the complex host-microbial interactions that drive inflammatory skin diseases. Our study investigated dermal versus epidermal microbiota in lesional and non-lesional skin of 37 adult atopic dermatitis (AD) patients and 37 healthy controls. Skin biopsies were partitioned into epidermal and dermal compartments, while serial tape strips collected the superficial epidermis. Bacterial communities were analyzed by cultivation, matrix-assisted laser desorption ionization time-of-flight mass spectrometry, confocal laser scanning microscopy, and metagenomic sequencing. We found that the effects of AD were evident across skin layers. The natural variation between skin layers and habitats diminishes in AD-affected skin, intensifying the impact of the microenvironment and host factors. A remarkably distinct dermal bacterial community was discovered among AD patients, being more conserved and providing a clearer difference between skin habitats, while the epidermis varied substantially. Importantly, comparisons between AD patients and controls revealed more genera differed when studying the dermal samples than the epidermal ones. *Staphylococcus*, *Corynebacterium,* and *Cutibacterium* genera differed with AD status across all samples, but *Prevotella* and *Mitsuokella* only differed in the dermis. In the dry and moist dermis, this translated into 14 and 61 gene pathways significantly varying with AD status, including many related to the biosynthesis of menaquinones (vitamin K2). These results suggest dermal sampling would allow for the role of the skin microbiome within AD pathogenesis to be better resolved since these communities are simpler and less prone to environmental contamination.

**IMPORTANCE:**

This study sheds light on the profound impact of skin microbiota's complex composition and distribution in atopic dermatitis (AD). The distinctive bacterial profile and activity, especially within the dermal skin compartment, vividly mirrored the cutaneous conditions in this inflamed microenvironment. The striking similarity in bacterial communities across different skin habitats in atopic skin underscores the high influence of atopic dermatitis—the genetic predisposition to an amplified immune response. This finding suggests that the dermal bacterial profile could be a valuable tool for longitudinally monitoring changes during the disease's relapsing phases, allowing for a precise categorization of patients into specific AD endotypes. Broadening the focus throughout the entire eczema-affected skin paves the way for treatments capable of modulating dermal biological factors, offering more effective management of AD. By further centering the interest in host-microbial interactions, we can refine personalized treatments, ultimately improving the lives of millions suffering from atopic dermatitis.

## INTRODUCTION

Human skin harbors a plethora of microorganisms, the composition of which varies between skin layers (1, 2) and habitats (3) in response to the local microenvironment (4). Though not completely understood (5), these microbial communities play an integral role in maintaining skin health (6, 7) and modulating skin disease development (8). Conversely, disruptions in the microbial skin balance, known as microbial dysbiosis, can reflect the severity of chronic, cutaneous inflammatory conditions, such as atopic dermatitis (AD) (9–19). In moderate-to-severe AD, dysbiosis is predominantly driven by the proliferation of *Staphylococcus aureus* (20, 21) and the simultaneous reduction in skin commensals (22–24). Despite this association, no clear causality between *S. aureus* colonization and AD pathology has been established, and eliminating *S. aureus* alone does not significantly improve AD outcome (25). This suggests that skin health is likely not determined by individual bacterial species but rather by a consortium of different microorganisms, or even a complex of multiple disorders with similar pathogenesis.

The vast majority of skin microbial studies have been confined to the outermost layer of the skin, the epidermis, often using superficial sampling methods, such as skin swabs. Beneath the epidermis, the dermis is colonized by a distinct subset of the epidermal microbiota (1). These dermal communities are more similar among healthy individuals than epidermal microbiota (1), as they are shaped by host-microbial interactions (26) and not confounded by transient contaminants (27). Thus, investigations into the taxonomic and functional variation of the dermal microbiota could provide valuable novel information and additional clarity into microorganisms’ role within AD pathogenesis.

**Fig 1:**
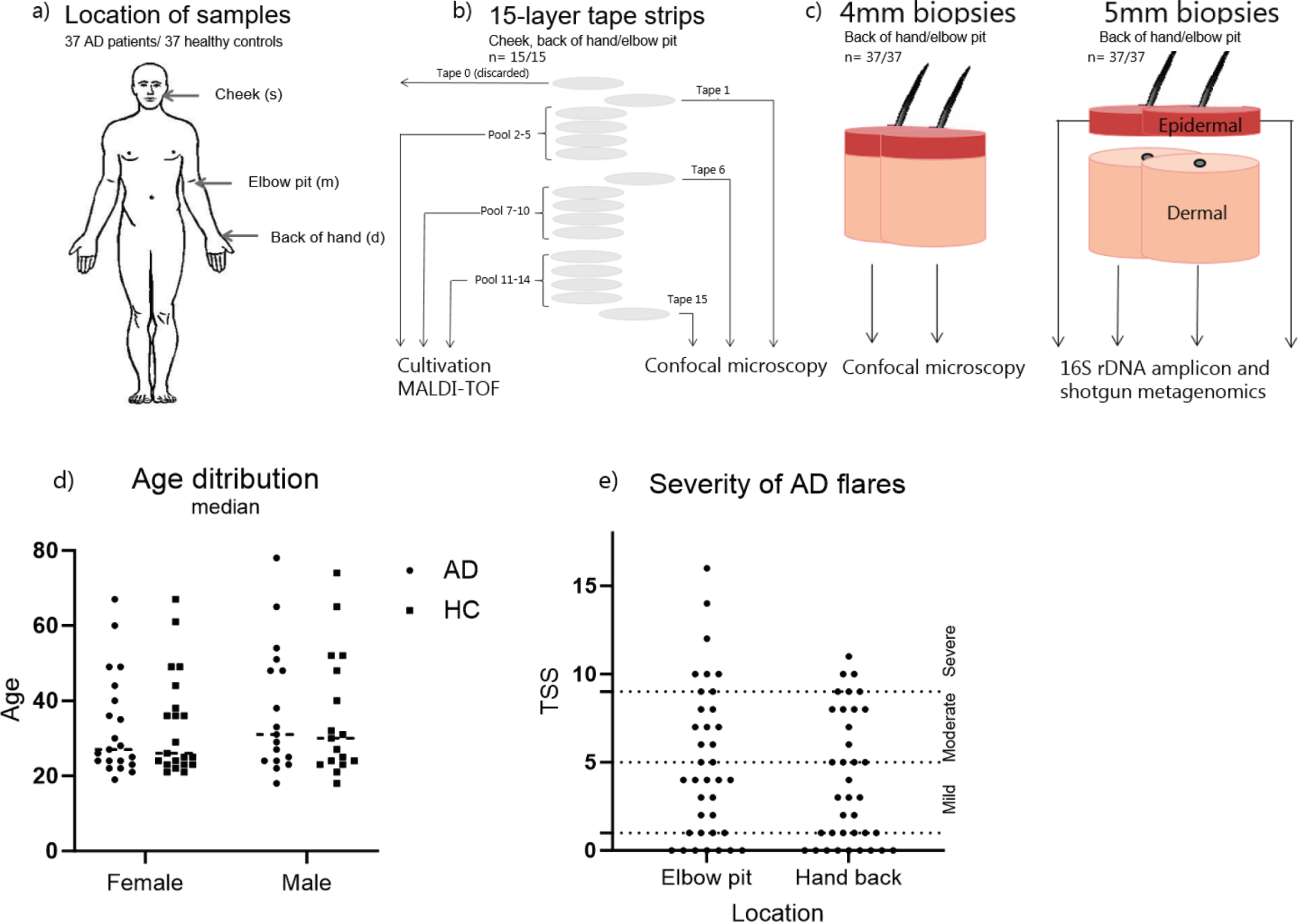
Schematic of participants, locations, samples, and analysis and metadata of participants in dot plots. Samples (a) were collected at three different habitats—dry (d), moist (m), and sebaceous (s) from the cheek, elbow pit, and back of hand, respectively. Fifteen consecutive tape strips (b) were collected for confocal laser scanning microscopy (CLSM) (tapes 1, 6, and 15), cultivation, and MALDI-TOF (pools 2–5, 7–10, and 11–14). Additionally (c), four biopsies, two from each elbow pit and back of hand, were collected for CLSM and metagenomics, respectively. The latter were divided into epidermal and dermal compartments before DNA extraction. (d) The distribution of age and sex is shown for the two groups of participants, atopic dermatitis (AD) and healthy (HC). Each dot represents one participant. The healthy participants were sex- and age-matched to the included AD patients. The average age of the participants was 34 years. (e) The total severity score (TSS) of eczema based on lichenification, excoriation, erythema, crusting/oozing, dryness/scaling, and edema (score of 0–3) is shown. Each dot represents the total TSS of the sampled area. Eczema categories: non-lesional = 0–1; mild = 2–5; moderate = 6–9; and severe = 10–18. TSS is shown with dotted lines.

This study determined whether microbial communities in dermal or epidermal compartments better differentiated AD patients from healthy controls across various skin habitats (dry, moist, and sebaceous). Consequently, the composition and distribution of the skin microbiota were explored between different compartments and habitats in AD and healthy skin. We sampled lesional and non-lesional AD skin from common eczema-prone areas (18): the back of the hand (dry), the elbow pit (moist), and the cheek (sebaceous) alongside corresponding areas from healthy individuals. To confirm the viable epidermal community composition and distribution, a multi-modal approach was utilized, incorporating CLSM, cultivation, and subsequent identification by MALDI-TOF mass spectrometry (28) to assess community structure from tape-stripped samples. Additionally, skin biopsies were examined by CLSM (29), and another set of biopsies was, after being partitioned into skin compartments, analyzed by 16S rRNA metabarcoding (1) (Fig. 1). While the functioning of the bacterial communities was attempted with shotgun metagenomics on biopsies, very few reads were microbial-derived (>0.1%). Therefore, the functioning profiles of the bacterial communities were compared using the 16S rRNA data.

## RESULTS

### Visualization of superficial and in-depth bacteria

**Fig 2:**
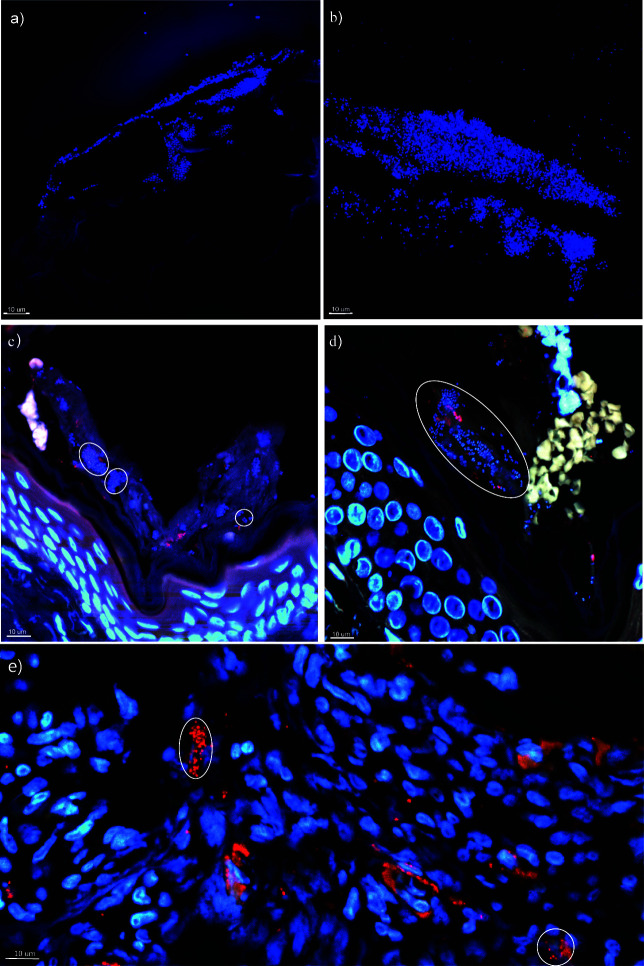
Visualization of bacterial aggregates and scattered single cells. Large bacterial aggregates (~1,500 µm in diameter) were detected on tape strips attached to the collected corneocytes in (a) AD moist skin habitat and (b) healthy sebaceous epidermal skin. Bacteria were stained blue by DAPI (DNA stain) and visualized using CLSM. Bacterial aggregates were detected in the stratum corneum of (c) AD and (d) healthy moist skin habitats and in (e) moderate lesional dry skin habitat. The aggregates detected in AD and healthy skin (white circles) sections were mostly <10 µm in diameter no matter the AD status. In the lesional skin, (e) bacterial aggregates were occasionally scattered deeper in the epidermis due to barrier abnormalities and structural changes. The paraffin-embedded and sectioned skin biopsies were stained by a universal bacterial Texas Red-conjugated PNA-FISH probe (mRNA stain) and DAPI. Human nuclei were stained blue by DAPI, while erythrocytes appeared yellow, and the surrounding tissue was green due to autofluorescence. The dual staining of the skin sections visualizes the bacterial metabolic activity (red = active, blue = inactive), showing more active bacterial aggregates in (e) lesional skin.

The cutaneous microbiota was heterogeneously distributed across the sampled skin layers. On skin tape strips, bacteria were found adherent to the collected corneocytes. Large bacterial aggregates (up to 1,500 µm in diameter) were detected in AD moist (lesional/non-lesional) and healthy (HC) sebaceous skin habitats (Fig. 2a and b). In contrast, bacteria were sparsely distributed in the dry skin habitat.

In skin sections, the microbiota was distributed as bacterial aggregates (10–15 µm in diameter) and as scattered single bacterial cells in the stratum corneum (Fig. 2c and d), at the infundibulum of hair follicles, and within hair follicles (data not shown). Aggregates were significantly more common (*P* = 0.015) in lesional skin (85%) compared to non-lesional skin (15%) and significantly more frequent (*P* = 0.005) in moist skin habitats compared to dry skin habitats, where single scattered bacterial cells were more common, regardless of AD status. Nevertheless, the overall distribution of aggregates was similar between AD and healthy skin with sizes less than 10 µm in diameter (30). However, several bacterial aggregates with high metabolic activity (red fluorescence of the probe) were detected in the deeper epidermis of severe lesional AD skin (Fig. 2e).

### Epidermal skin microbiota cultivated from skin tape strips

**Fig 3:**
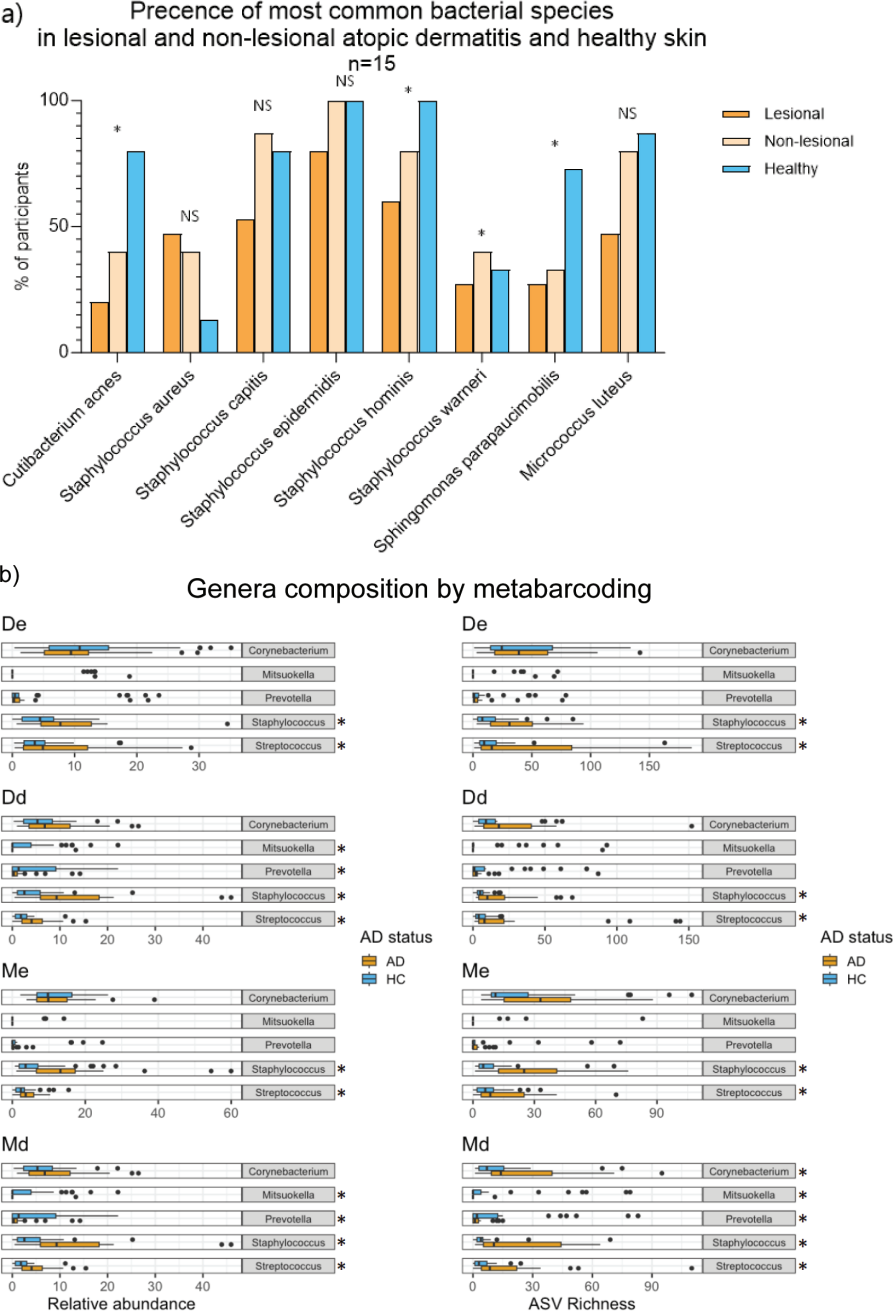
Specific species and genera composition by cultivation and 16S amplicon sequencing. (a) A bar chart shows the most common cultivated species in all skin groups for AD lesional, (black) non-lesional (gray), and healthy skin (white). The statistical difference (*χ*-square) is shown above the bars. (b) Box plots visualizing significant differences in relative abundance/ASV richness of abundant genera between AD patients and healthy controls by 16S rRNA amplicon sequencing. Sample types: De = dry epidermal, Dd = dry dermal, Me = moist epidermal, and Md = moist dermal compartment. Asterisks denote significant differences. *Pseudomonas* showed significant differences in ASV richness within the Me samples but was removed for ease of reading.

Regardless of skin tape strip layers, skin habitats, or lesion severity, the composition of the epidermal microbial community differed significantly (*P* = 0.01) between AD (lesional and non-lesional) and HC skin (Fig. 3; Fig. S2). Bacterial CFUs were significantly increased in AD compared to HC in both dry (*P* = 0.013) and moist (*P* = 0.033) skin habitats (lesional and non-lesional combined) but not in the sebaceous skin habitat (non-lesional) (Fig. S2). *S. aureus* was significantly more prevalent (*P* = 0.020) in the AD epidermis (60% in lesional and non-lesional) than in HC (13%) (Fig. S2, Table S8). Conversely, *Cutibacterium acnes* was significantly less common in lesional AD skin (20%, *P* = 0.003) and non-lesional AD skin (47%, ns) compared to HC epidermal skin (80%). Meanwhile, *Staphylococcus epidermidis* was ubiquitous in all skin samples. None of the negative control tapes produced CFUs, indicating no issues with contamination.

### Dermal and epidermal microbiota of AD and healthy skin using 16S amplicon sequencing

Bacterial communities were profiled in partitioned epidermal and dermal biopsies from moist and dry skin habitats of AD and HC participants. We initially tested the effect of lesion status (lesional vs. non-lesional) on amplicon sequence variant (ASV) richness and community composition in AD samples and found no relevant/significant effect [composition: *R*^2^ = 0.011, *P* = 0.138 and richness: Akaike information criterion (AIC) = 1,132, *P* = 0.748]. We also tested the effects of AD severity (none to severe) on the bacterial communities in AD skin from the two locations, finding no significant differences in ASV richness or community composition (dry habitat—composition: *R*^2^ = 0.019, *P* = 0.405 and richness: AIC = 2.79, *P* = 0.095; moist habitat—composition: *R*^2^ = 0.022, *P* = 0.172 and richness: AIC = 3.39, *P* = 0.066). Therefore, AD samples (lesional and non-lesional) were pooled together in comparisons against the HC samples.

Initial analysis of the data identified significant effects of AD status (*R*^2^ = 0.008, *P* = 0.002), skin compartments (epidermal vs. dermal) (*R*^2^ = 0.021, *P* = 0.001), and skin habitats (moist vs. dry) (*R*^2^ = 0.007, *P* = 0.013) on community composition and ASV richness (Fig. S3, Table S1). Therefore, samples were divided into four skin groups, namely, dry-epidermis, dry-dermis, moist-epidermis, and moist-dermis, to further explore the effects of AD without the confounding influence of skin depth and habitat (Table S2).

AD had a significant effect on the bacterial composition within each of the four skin groups (Fig. S4), accounting for approximately 3% of community variation in each group, with the effect being slightly higher in the moist dermis skin group (3.7%) (dry dermis*—R*^2^ = 0.029, *P* = 0.009, dry epidermis*—R*^2^ = 0.028, *P* = 0.010, moist epidermis*—R*^2^ = 0.029, *P* = 0.004, and moist dermis*—R*^2^ = 0.037, *P* = 0.001). In the dry dermis, AD patients had significantly higher richness (284 ± 180) compared to HC participants (223 ± 113) (AIC = 597.7, *P* = 0.010). No significant effect of AD on richness was observed in the other skin groups (Table S1). Between-subject variation in richness was considerably lower in AD compared to HC skin.

**Fig 4:**
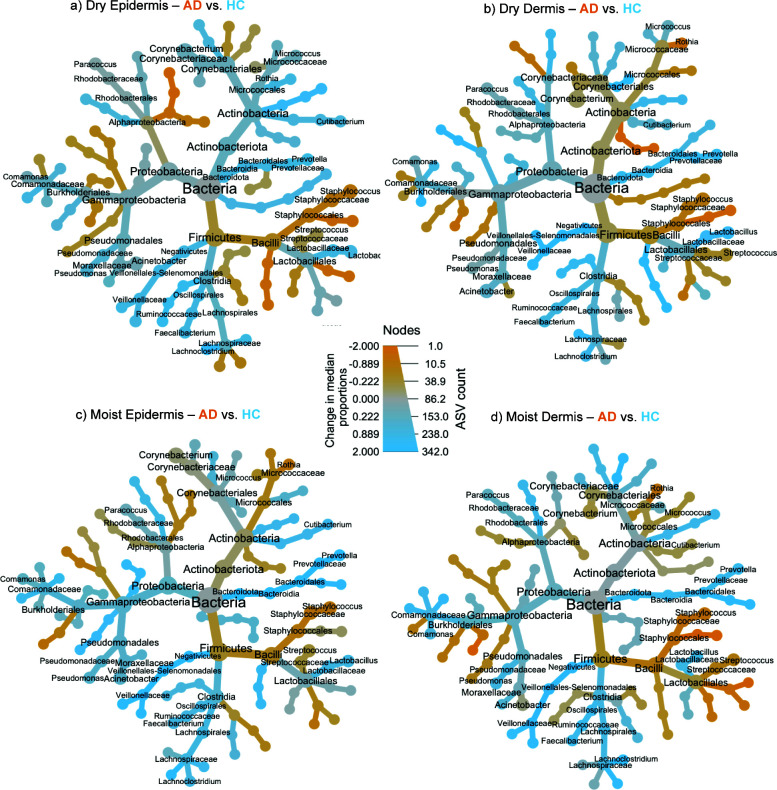
Bacterial specificity in skin compartments in AD and healthy controls. Heat trees of ASVs differing between the bacterial community of AD (orange) and healthy (blue) within the four skin compartments: (a) the dry epidermis, (b) dry dermis, (c) moist epidermis, and (d) moist dermis. Gray nodes are equally represented in both comparing groups. After correction for multiple comparisons, no ASVs statistically varied in relative abundance between AD and healthy, while many ASVs remained significantly different within the dermal compartment.

Microbial profiles between AD and HC skin, as well as between the epidermal and dermal compartments of AD, were visualized as taxonomic heat trees (Fig. 4; Fig. S4). The analysis revealed consistent differences in the communities associated with AD, with particularly pronounced variation in the *Staphylococcus*, *Corynebacterium*, and *Cutibacterium* genera (Fig. 4; Fig. S5, Table S4 and S5). Consequently, significant differences in relative abundances and ASV richness were tested at the genus level in each skin group. *Staphylococcus* and *Streptococcus* consistently showed significantly higher ASV richness and relative abundances in AD samples compared to HC across all skin groups (Fig. 3B), except for the moist epidermal compartment (Table S4). Interestingly, dermal communities were better at identifying significantly varying genera between AD and HC skin, with equal to or greater numbers of genera varied with AD status. For example, the *Prevotella* and *Mitsuokella* genera were found to have significantly higher relative abundance and ASV richness in the dermis but did not vary in either measure in the epidermis (Fig. 3b).

Significant differences in the abundance of individual AD-associated ASVs were also investigated for each skin group. Although many were identified, only two remained significant after correcting for multiple comparisons (using false-discovery rate; Table S5). Both were found within the moist dermal skin group: a *Staphylococcus* species (ASV1), which significantly increased from an average relative abundance of 0.14% in the healthy participants to 0.92% in AD participants, and a *Gemella* species (ASV34), which significantly increased in relative abundance within AD (0.27%) compared to HC participants (0.04%).

### Functional analyses

**Fig 5:**
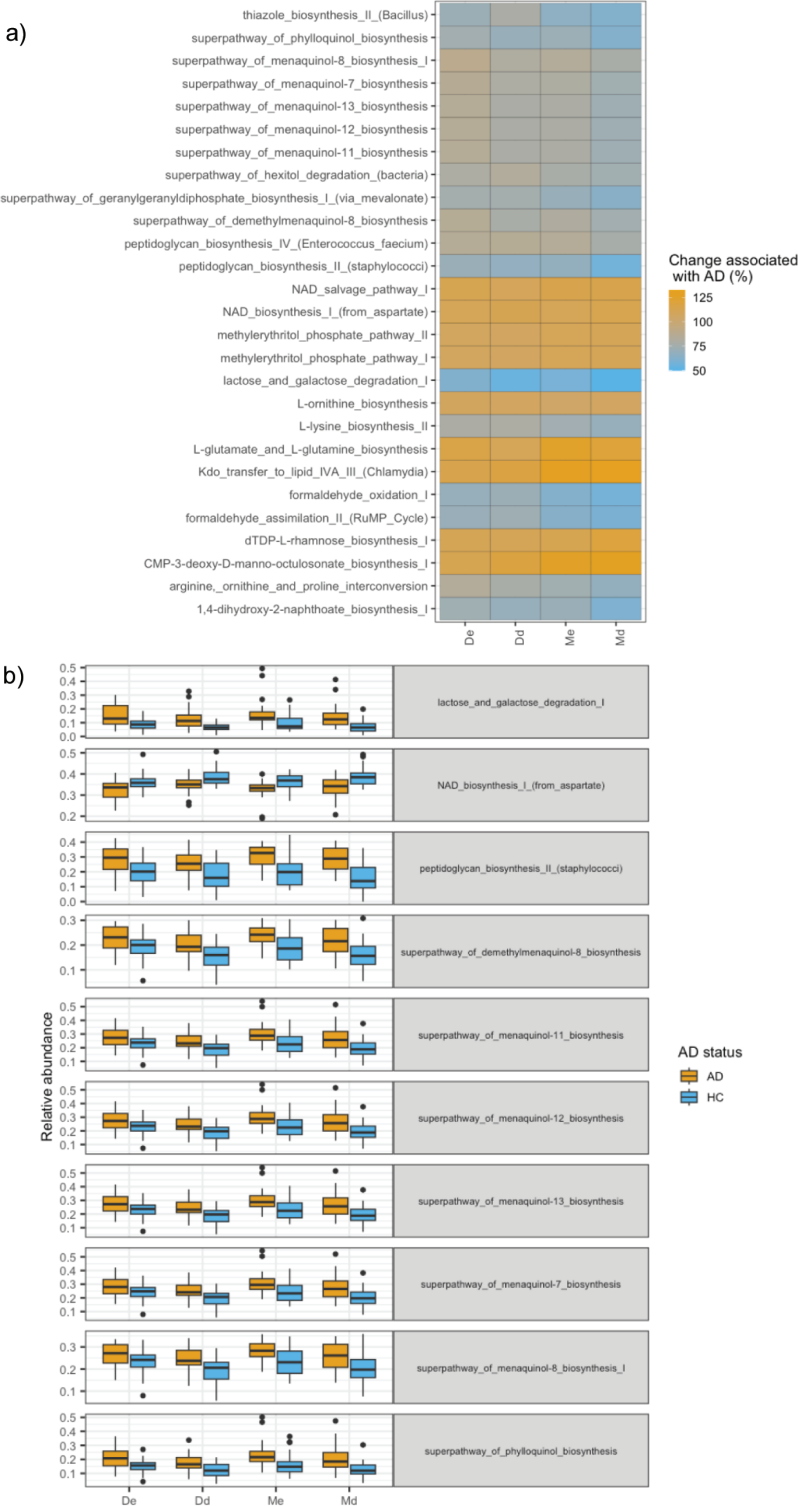
Significant variation in gene functions of the bacterial communities in AD and healthy controls. (a) A total of 27 pathways significantly differed in two or more skin groups. These 27 pathways were plotted, with their change in relative abundance associated with AD mapped (orange is AD; blue is controls). Pathways consistently differed between skin groups; however, the dermal and/or moist environments best differentiated AD skin from healthy controls. (b) Ten pathways significantly differed in all three skin groups (none varied in the dry epidermis). Nine of the 10 pathways were consistently higher in AD patients than healthy controls.

The shotgun metagenomics produced fewer than 1,000 reads per sample after removing human reads. Due to the limited number of microbial-derived reads, gene pathways were annotated using metabarcoding data, which have previously been found to provide useful functional profiles for the bacterial communities in humans (31). Interestingly, many significant differences were observed in the functional profile of the skin bacteria compared to the taxonomic comparisons (Table S6). A larger difference in functional capacity (in contrast to taxonomic variation) was observed in the moist relative to dry habitat and within dermal compared to epidermal communities when comparing AD and HC skin. Specifically, in the dry habitat, no pathways significantly differed in the epidermal compartment, but 14 were found to vary within the dermal compartment. In the moist habitat, 25 and 61 pathways significantly varied in the epidermal and dermal compartments, respectively. Individual pathways showed consistent differences between AD and HC across skin layers (i.e., when significantly higher expression was found in AD moist epidermis compared to HC, a similar pattern was observed in the remaining skin groups, even if not significant) (Fig. 5). Three of the four skin groups showed significant differences in the expression of the same 10 pathways (the dry epidermis had no varying pathways). Seven of the pathways were associated with menaquinol, all of which were significantly more expressed in AD than in HC skin. The remaining pathways were associated with lactose and galactose degradation, which were highly expressed in AD skin, and NAD biosynthesis, which had higher expression in the HC skin.

## DISCUSSION

Our approach of dividing biopsies into skin compartments from diverse skin habitats provided detailed insight into the unique microbiota of AD relative to HC skin (Fig. 3b). It was revealed that dermal samples combined with traditional epidermal sampling provide new insights and nuances into the role of skin microbiome in AD.

The effect of AD on skin microbiota was especially clear when examining the dermal samples, where additional genera varied significantly with AD status. The reduced richness of dermal bacteria supports our hypothesis that dermal sampling avoids transient microorganisms, and in severe AD skin, it may offer a clearer correlation between microbial composition and AD progression. The dermal microbiota was generally more conserved between habitats than the epidermal microbiota, providing deeper insights into the microbial shifts associated with AD at both taxonomic and functional levels. Dermal microbiota is shaped primarily by the host’s immune response and the local microenvironment, offering a more accurate representation of the biological mechanisms driving AD. In contrast, epidermal bacteria influenced not only by immune responses but also by topical treatments and environmental factors better reflect individual variations and lifestyles among AD patients. This distinction underscores the complementary roles of dermal and epidermal microbiota in AD: the former provides insights into disease pathology, while the latter highlights individual treatment responses and environmental interactions. Together, they establish a new framework for understanding AD that can guide and improve therapeutic approaches.

Consistent with our previous studies showing a distinct dermal healthy community (1) and an AD-specific profile (32), we found the AD profile was significantly different across both skin compartments and habitats, where differences were associated with skin layer (1, 32), skin habitat (3), and AD status (33). In addition, our findings suggest that while the microbial composition is similar between lesional and non-lesional skin, there are considerable differences in microbial abundance. Previous research linked these differences to an increased abundance of *S. aureus* in lesional skin (33, 34), further supporting the idea that AD has a unique microbial composition that shifts during flare-ups, particularly with the proliferation of *Staphylococcus* spp*.*

We observed alterations in the bacterial community composition and function across all skin groups, indicating a widespread and consistent relationship between AD and skin bacteria. For instance, we noted increased abundance and ASV richness of both *Staphylococcus* and *Streptococcus* species in all AD skin groups (Fig. 4). The *Prevotella* genus, significantly varying between AD and HC dermis, was previously found in lower abundance in the AD epidermis compared to controls (35). It is further associated with other skin diseases, such as hidradenitis suppurativa, where it is correlated to early inflammation (36, 37) and is frequently found in HS tunnels (38). Interestingly, the gut-associated *Mitsuokella* genus has not been previously connected with AD skin, and, here, we found significant decreases in both skin habitats, but only in the dermis. The differences in microenvironment in skin layers and habitats have been routinely shown to lead to different community assemblies (32), and they must be considered before fully understanding the microbiome’s role in AD pathogenesis. Notably, the high similarity of the dermal microbiota across habitats supports that the underlying microenvironment is also more stable in deeper AD skin layers (39). Studying dermal microbial communities may help mitigate challenges like inter-individual variation and differing sampling locations, which complicate understanding the interaction between the skin microbiome and AD pathogenesis.

Our study revealed significant differences in microbial pathways between AD and HC skin, particularly in the biosynthesis (of the precursors of) menaquinone/phylloquinone (40) (Fig. 5). In AD patients, vitamin K2 attenuates activated T-cell immunity (41), but the menaquinone biosynthesis decreases during treatment (42), which could suggest a role in AD pathogenesis. Additionally, decreased functional capacity for NAD biosynthesis could indicate less involvement of skin bacteria in the host metabolism, DNA repair, and aging process influenced by the NAD cofactor (43). The increased functional capacity of lactose and galactose could indicate enriched competing capabilities of the AD microbiome for the potentially limited sugar availability (24). Correspondingly, increased peptidoglycan production by *S. aureus* may exacerbate the Th2 immune response (44), a hallmark of AD’s impaired immunity.

Cultivation of the epidermal microbiota in our study revealed substantial changes in the AD community (Fig. 3a), much larger than what was observed through 16S rRNA amplicon metabarcoding. The notably increased presence of *S. aureus* in AD skin, along with differences in species composition within specific genera, complements the study of Fyhrquist et al., suggesting that species- and strain-level variation within key taxa is associated with AD (24), even in non-lesional, mild, and moderately affected skin (22). Concurrently, we found that the microbial genus profiles were similar between lesional and non-lesional skin, whether assessed by cultivation or sequencing, and did not differ according to eczema severity. This suggests that while the abundance of specific bacteria fluctuates during AD flare-ups, the overall microbial community remains stable over time, confirming recent findings in AD skin (24) and gut microbiome (42). This stability may be linked to pathophysiological cutaneous conditions, that is, the dysfunctional skin barrier (18, 45), abnormal lipid content (46), and elevated pH (47).

Interestingly, while *C. acnes* dominated the healthy sebaceous skin, it was replaced by *Staphylococcus*, *Streptococcus*, and *Corynebacteria* in AD patients. Despite these shifts, the total CFUs were not significantly different between AD and healthy skin, suggesting saturation of the environment. These species-level shifts across a wide range of genera highlight the need to examine the entire microbial community further rather than focusing solely on *Staphylococcus*.

This study also confirmed the presence of bacterial aggregates attached to the corneocytes (48) of stratum corneum and the heterogeneously distributed small bacterial aggregates and scattered single cells on the outermost epidermal layers and within the depth of hair follicles (29, 30, 49). Aggregation is a natural growth form of bacteria also found in healthy skin (29, 30). However, despite the impaired tight junctions in AD skin associated with increased susceptibility to pathogens and infections (50), microbial penetration into deeper skin layers was only observed (51) in severe lesions. This suggests that the thickened stratum corneum in mild to moderate AD may help to protect the skin by shedding accumulated bacteria.

Methodologically, our study highlighted the value of combining data sets in providing insight into bacterial composition and distribution. This is invaluable for understanding AD, as the bacterial community is expected to change with AD, predominantly driven by *Staphylococcus* spp., including *S. aureus*, while overall bacterial biomass is expected to increase in the outermost layers but decrease with increasing skin depth (2). However, while culturing is limited to culturable taxa, metabarcoding lacks the taxonomic resolution to distinguish closely related taxa (52), such as *Staphylococcus* (48). Results did not improve, even when we re-ran analyses using a custom *Staphylococcus*-specific database for taxonomic assignments. Meanwhile, shotgun metagenomics (and metagenomic-assembled genomes) allows specific strains of *S. aureus* to be linked to more severe AD and *S. epidermidis* to healthy controls (22). However, even ultra-deep sequencing could not yield a viable number of microbial-derived reads from the skin biopsies, as the host DNA was too dominant.

The sampling of identical skin locations in all participants strait-lined the data; however, it also entailed a wide variation in the eczema scores and limited the sample sizes of the subgroups. Although incorporating the sebaceous skin habitat in the metagenomic analysis might have given us a better understanding of the possible dysbiosis in sebaceous dermal AD skin and maybe even of taxa not cultivated, we did not collect biopsies from the patients’ cheeks due to ethical considerations. Furthermore, the lack of alignment in the patients’ previous treatment may have affected the microbiota and the microenvironment of the skin or introduced more stochastic variables to the data set, but this was also accepted to accommodate the individuals suffering from AD.

Relatively recently developed long-read amplicon sequencing could be a potentially fruitful avenue for further exploration. For example, the entire 16S rRNA region could be sequenced to provide higher taxonomic resolution while also incorporating an amplification step to overcome the high host-to-microbe DNA ratio (53). This could be coupled with culturing and/or digital droplet PCR to provide quantitative information. Furthermore, future projects should increase the number of participants for cultivation to account for high individual variation in epidermal microbiota. From a clinical perspective, future studies would benefit from a longer washout period before sampling to avoid the influence of prior treatments (54). Despite the invasive nature of biopsies compared to swabs or tape-stripping, studying the dermal microbiota could provide deeper insights into the links between AD pathogenesis and microbial dysbiosis.

The effects of AD often seem confined to the epidermis, which is why most research has historically focused on this outer layer. However, our deeper investigation strongly supports the idea that the signs observed in the epidermis are, to some extent, surface reflections of deeper interactions within the dermis. These superficial signs are likely exacerbated or alleviated by external factors, explaining why current AD treatments, such as topical corticosteroids and emollients that primarily target the epidermis, provide relief, though only temporarily. Shifting the focus to the deeper skin layers represents a true paradigm shift, unlocking promising prospects for innovative AD treatments that penetrate beyond the surface to modulate dermal biological factors, offering a more informative approach to managing chronic AD and preventing flare-ups.

### Conclusion

Significant differences in the microbial composition, with more *Staphylococcus* and fewer *Cutibacterium* species in AD relative to healthy epidermal skin, were confirmed by both cultivation and metabarcoding. Meanwhile, the effects of AD were detectable in both the dermis and epidermis, but more pronounced in the dermis, displaying a unique bacterial profile distinct from the healthy assembly. The dermal communities were more conserved taxonomically across all samples, but by studying the dermal compartments, we were better able to delineate the AD core microbiota, with more specific genera and genes found to significantly differ between AD and healthy participants. Furthermore, the difference in bacterial functional profiles between AD and healthy skin was also greater at the dermis than at the epidermis. Therefore, future studies should consider utilizing the microbiome from all skin layers and subtracting confounding epidermal taxa to better subdivide patients into endotypes and improve personalized treatment.

## MATERIALS AND METHODS

### Location of performance

Samples were collected at the Department of Dermatology and Allergy, Herlev-Gentofte Hospital. Sample processing and data analyses were performed at the Department of Immunology and Microbiology, Department of GeoGenetics, University of Copenhagen, and Department of Clinical Microbiology, Rigshospitalet, Denmark.

### Participants and sampling

Participants diagnosed with atopic dermatitis (AD) according to the diagnostic criteria (55, 56) and healthy controls were recruited in the clinic and at forsoegsperson.dk. The inclusion criteria were that participants were legally competent individuals over 18 years old. Additionally, for AD participants, active eczema on the back of the hand and/or in the elbow pit should be present. Meanwhile, participants who were pregnant had used topical corticosteroids within 48 h, moisturizing cream within 24 h, or oral or topical antibiotics 4 weeks before sampling were excluded. Exclusion criteria for healthy participants included those with any skin disorders, active infections, or wounds in the sampling area. Furthermore, the participants were instructed to avoid chloride swimming pools 24 h before, not bathe or use cosmetics on the day of sampling, and not to use soap or ethanol on the sample sites (including hands) 1 h before sampling. Thirty-seven Caucasian participants with AD (male:female ratio, 17:20) with an average age of 34 years (18–78 years) and 37 age- and sex-matched healthy controls were included after giving written consent (Fig. S3, Table S7). Total severity score (TSS) was assessed by the same two clinicians trained as assessors according to the guidelines of the International Eczema Council (57). The AD sample sites were assessed based on lichenification, excoriation, erythema, crusting/oozing, dryness/scaling, and edema with a score of 0–3 in each category (58). AD skin was considered non-lesional when the TSS was one or below, mild when between 2 and 5, moderate when between 6 and 9, and severe when 10 or above. TSS is a local assessment similar to target lesional severity score (TLSS) (59), of which categories and scales also govern the widely used severity scoring of atopic dermatitis (SCORAD) and eczema area and severity index (EASI) (58).

In 15 AD and 15 HC participants, 16 layers of tape strips DS100 D’Squame disc tapes (Monaderm, Monaco) were collected (28) at the back of the hand, elbow pit, and cheek. Negative control tape strips were collected and handled alongside the samples. A tape strip was placed on the skin, and markings were made with a cosmetic pencil on the surrounding skin to mark the position. This first tape strip (no. 0) was discarded. The following tape strips were positioned and sampled within the markings, and a uniform pressure of 225 g/cm^2^ was provided on the tape strips for 15 s with a spring-loaded device (Clinical Derm, Dallas, TX, USA) before collecting the sample. Tape strip numbers 1, 6, and 15 were separately stored in 2 mL cryotubes, with the glue side facing the tubes’ inner lumen. Pools of tape strip numbers 2–5, 7–11, and 11–14 were stored in 5 mL cryotubes and positioned along the sides, with the glue side facing the inner lumen of the tubes. All tape strips were further processed the same day.

Two punch biopsies (4 and 5 mm in diameter) were collected by local anesthesia (lidocaine-adrenalin 20 mg/5 µg per mL) from both the elbow pit (lateral part of antecubital fossa) and back of the hand (dorsal side between the thumb and the index finger) from all participants (Fig. 1). One skin biopsy (4 mm) was fixated (29), while the other (5 mm) was divided into dermal and epidermal compartments (1) and stored at −80°C.

### Cultivation and bacterial identification

The 1,440 tape strips were distributed into 540 samples: 18 for each participant. To examine epidermal layers, tape pools 2–5, 7–10, and 11–14 were applied 3 mL of 0.9% saline, degassed, and sonicated for 5 min in an ultrasonic bath (Branson 2510, Sigma-Aldrich, St. Louis, MO, USA). The fluid was diluted in a 10-fold dilution series, plated on fastidious anaerobic agar with horse blood (Thermo Fischer Scientific, Waltham, MA, USA), blood agar, and chocolate agar (SSI Diagnostica, Hillerød, Denmark), and incubated under anaerobic (Concept 400, LAF Technologies, Boronia, VIC, Australia) and aerobic conditions in atmospheric air and with CO_2_ (Sanyo incubator, Osaka Japan) at 37°C for 1–3 days. Colonies were quantified in CFU, isolated, and stored in Luria Bertani broth containing 20% glycerol at −70°C (28) (Fig. 1, Tables S8 and S9). Negative control tape strips were cultivated along the samples to rule out contaminations. The bacterial isolates were identified by matrix-assisted laser desorption/ionization—time-of-flight mass spectrometry (MALDI-TOF ms) using MALDI Biotyper (Bruker, Bremen, Germany). The isolated cultures were applied on a target plate (MSP 96 polished steel BC, Bruker), and 1 µL of an energy-absorbent matrix HCCA (a-cyano-4-hydroxycinnamic acid, Bruker) was added. Samples were analyzed using the MTB Compass (v4.1) and Flexcontrol (v. 3.4) software and the BDAL standard library. The acceptance level of the ID score was >2.0 according to the manufacturer manual. At lower scores, the isolates were re-analyzed.

### Staining and confocal microscopy

Tape strip numbers 1, 6, and 15 were stained for microscopy by applying 3 µM 4′,6-diamidino-2-phenylindole (DAPI) (Life Technologies, Carlsbad, CA, USA) to a microscopic coverslip and placing the tape on the top. The coverslip was glued to a microscope slide using clear nail polish and examined by confocal laser scanner microscopy (CLSM).

Two to four consecutive perpendicular tissue sections (4 µm) per sample were deparaffinized in Xylene (2 × 5 min), 99% ethanol (2 × 3 min), 96% ethanol (2 × 3 min), and Milli-Q water (3 × 3 min). A universal bacterial-specific PNA-FISH Texas Red-conjugated probe (AdvanDx, Woburn, MA, USA) targeting the 16S ribosomal RNA was applied to the tissue sections and incubated at 55°C for 90 min, after which surplus dye was removed using a 55°C wash buffer (AdvanDx) for 30 min. The tissue sections were counter-stained with 3 µM DAPI for 15 min at RT, rinsed with Milli-Q water, and subsequently mounted with mounting media (Prolong Gold, Life Technologies), upon which a cover slide was sealed with clear nail polish (29, 30).

Tape strips and tissue sections were examined by CLSM (LSM710, Zeiss, Oberkochen, Germany) using a 63×/NA 1.4 Plan-APOCHROMAT oil objective (Zeiss) at the excitation wavelengths of 405, 488, and 594 nm and emission wavelengths of 410–483 nm (blue), 492–589 nm (green), and 599–690 nm (red) with the beam-splitters MBS 488/594 and MBS InVin 405 (Zeiss). Representative images were taken using the software ZEN black (Zeiss) and deconvoluted using the interactive microscopy image analysis (Imaris) version 9 (Bitplane, Zurich, Switzerland) (30). Semi-quantifying the detection of bacterial cells or aggregates was defined as 0 = no bacteria detected, 1 = aggregates < 5 µm (diameter) or <10 single scattered bacterial cells, 2 = 5–10 µm aggregates or >10 single cells, 3 = 10–50 µm aggregates, and 4 =>50 µm aggregates (30).

### Amplicon and shotgun metagenomics

DNA extraction was performed as per Bay et al. (1) at the Centre of GeoGenetics, Copenhagen, Denmark. Skin biopsies divided into dermal and epidermal compartments (1) and extraction controls were lysed for 10 min at 30 Hz using a TissueLyser (Qiagen, Hilden, Germany). DNA was extracted using the MO BIO PowerViral environmental RNA/DNA isolation kit (Qiagen, Hilden, Germany) according to the protocol of the manufacturer. The PCR amplification, library preparation, and metabarcoding were produced at Novogene Company Limited, Cambridge, UK. Region V3–4 of the 16S rRNA gene was amplified using 341F and 806R primers with barcodes. PCR reactions were carried out in 15 µL of Phusion High–fidelity PCR Master Mix (Thermo Scientific, Waltham, MA, USA), with 0.2 µM of each primer and 10 ng template DNA. The PCR program consisted of 98°C for 60 s and 30 cycles of 98°C for 10 s, 50°C for 30 s, and 72°C for 30 s, finishing with 72°C for 5 min. PCR products were purified using magnetic bead purification, and target bands were recovered (Novogene confidential). Libraries were generated with indexes using the NEBNext Ultra II DNA Library Prep Kit (New England Biolabs, Ipswich, MA, USA), while Qubit, RT-PCR, and the Bioanalyzer verified the concentration and size distribution. Libraries were sequenced on the Illumina HiSeq NovaSeq 6000 (250 bp paired end) platform with a minimum of 50,000 raw reads per sample. Negative controls from DNA extraction and PCR negatives were included in each batch and analyzed along with the samples. Shotgun metagenomic data were produced for a subset of 100 biopsies by Novogene (UK) using the NovoSeq X platform. In total, approximately 21 million reads per sample were produced. Human reads were removed using Diamond, whereby it was determined that too few reads remained to proceed (>1,000 per sample).

### Bioinformatics and statistics

Sequencing data were processed using DADA2 as per Barnes et al. (60). Briefly, demultiplexed reads were quality-filtered to remove reads with any ambiguous bases and below 100 bp. Reads then underwent error correction, merging of paired ends, dereplication, and chimera removal using DADA2’s native algorithms using default settings. Taxonomy was assigned using the Silva 138 database using the RDP classifier (within DADA2). The dermal microbiome is very low and therefore highly susceptible to contamination. Hence, a highly stringent approach to contamination removal was performed. Initially, all 27 negative controls were processed; however, only eight produced any reads that remained after processing. After which, the ASVs present in four or more of these controls (extraction or PCR blacks) were removed from the entire data set, accounting for 15 ASVs. Additionally, reads not assigned to Bacteria or Archaea and, lastly, any samples with fewer than 2,000 reads remaining were removed. In total, 12,778,798 passed through the DADA2 process, and 9,688,272 reads remained after further filtering spanning across 8,677 ASVs. This was a mean average of 38,143 (±31,944) per sample. Finally, reads underwent a fourth root transformation and normalization to relative abundances for downstream analyses.

Due to the failure of shotgun metagenomics to produce a usable number of reads, the functional profile of the bacterial community was predicted using Tax4Fun2 (61), with these methods providing functional insights into the bacterial community that can be used cautiously (61, 62). This function annotation was performed by parsing the relative abundance table and a table of taxonomy through the Tax4Fun *R* package using the Tax4Fun2 reference database (version 2). The 16S data were normalized by copy number and had a minimum identity to reference of 97%.

Differences in CFU were analyzed by two-way analysis of variance (ANOVA). Metabarcoding data were analyzed for species composition (beta diversity) using PERMANOVA. A *χ*^2^ test was used to analyze the contingency of *S. aureus* in AD skin. Statistics were calculated, and plots were made using R (version 2.14.9) and Prism 9.3 (GraphPad, Inc., La Jolla, CA, USA) software.

## Data Availability

The raw and sequenced data are freely available for download at the accession ID PRJNA1251692 at NCBI SRA.

## SUPPLEMENTAL MATERIAL

Fig. S4 msphere.00156-25-s0001.pdf Additional taxonomic trees.

Fig. S1 to S3 msphere.00156-25-s0002.pdf Scattered, box, and bar plots of additional data.

Supplemental tables msphere.00156-25-s0003.xlsx Tables S1 to S9.

ASM does not own the copyrights to Supplemental Material that may be linked to, or accessed through, an article. The authors have granted ASM a non-exclusive, world-wide license to publish the Supplemental Material files. Please contact the corresponding author directly for reuse.
