# Supplementary material for: Unique dermal bacterial signature differentiates atopic dermatitis skin from healthy
Source: mSphere. 2025 May 9;10(6):e00156-25. doi: 10.1128/msphere.00156-25 (PMC12188723; doi:10.1128/msphere.00156-25)
Supplement: Fig. S4 — Additional taxonomic trees. [file msphere.00156-25-s0001.pdf]

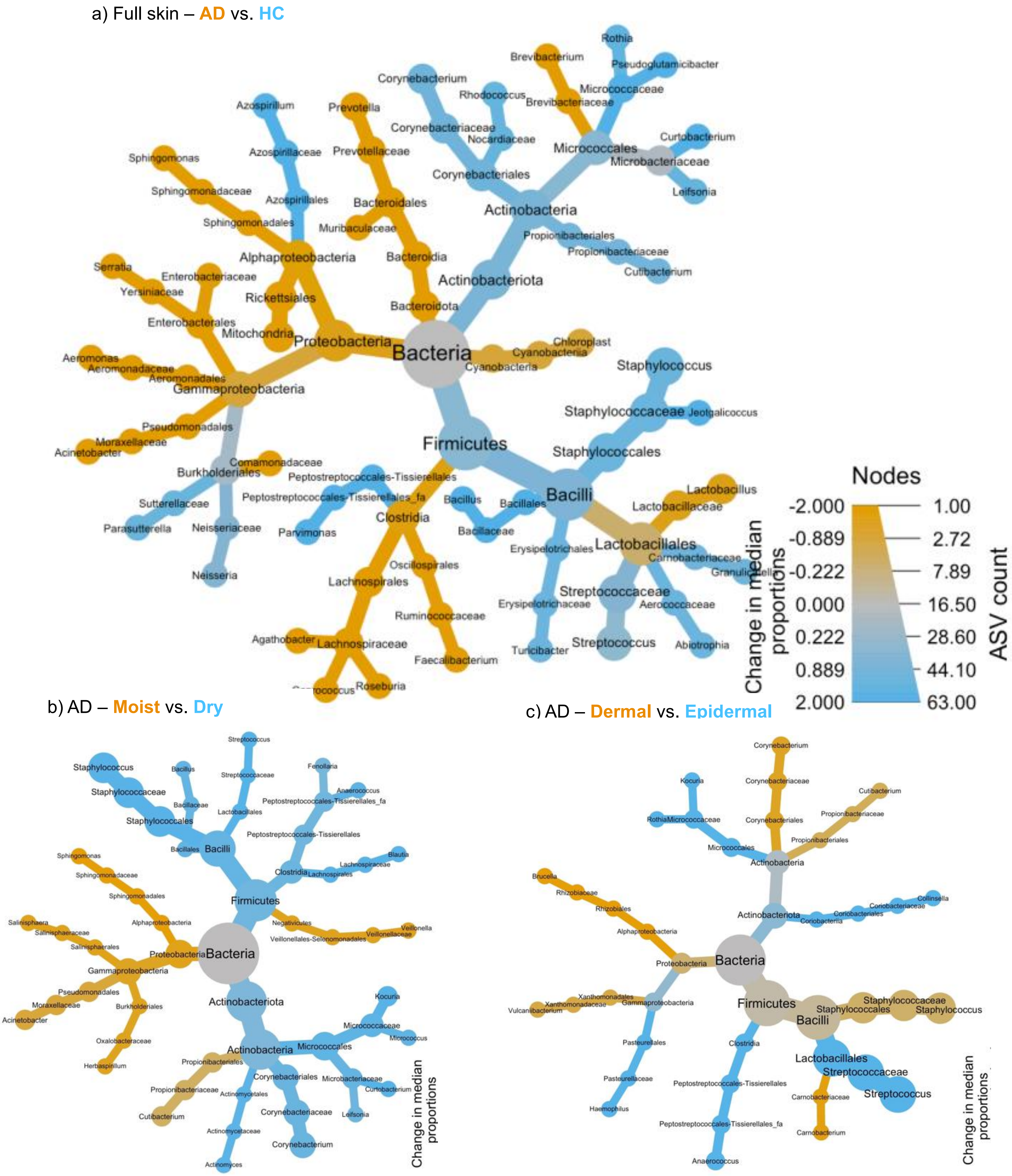

a) Full skin – AD vs. HC
Bacteria
Proteobacteria
Actinobacteriota
Actinobacteria
Firmicutes
Bacilli
Cyanobacteria
Chloroplast
Bacteroidota
Bacteroidia
Bacteroidales
Prevotellaceae
Prevotella
Muribaculaceae
Alphaproteobacteria
Gammaproteobacteria
Rickettsiales
Mitochondria
Sphingomonadales
Sphingomonadaceae
Sphingomonas
Azospirillales
Azospirillaceae
Azospirillum
Enterobacterales
Enterobacteriaceae
Yersiniaceae
Serratia
Aeromonas
Aeromonadaceae
Aeromonadales
Pseudomonadales
Moraxellaceae
Acinetobacter
Burkholderiales
Comamonadaceae
Sutterellaceae
Parasutterella
Neisseriaceae
Neisseria
Corynebacteriales
Corynebacteriaceae
Corynebacterium
Nocardiaceae
Rhodococcus
Micrococcales
Micrococcaceae
Rothia
Pseudoglutamicibacter
Brevibacteriaceae
Brevibacterium
Microbacteriaceae
Curtobacterium
Leifsonia
Propionibacteriales
Propionibacteriaceae
Cutibacterium
Staphylococcales
Staphylococcaceae
Staphylococcus
Jeotgalicoccus
Bacillales
Bacillaceae
Bacillus
Lactobacillales
Lactobacillaceae
Lactobacillus
Carnobacteriaceae
Streptococcaceae
Streptococcus
Aerococcaceae
Abiotrophia
Erysipelotrichales
Erysipelotrichaceae
Turicibacter
Clostridia
Peptostreptococcales-Tissierellales
Peptostreptococcales-Tissierellales_fa
Parvimonas
Lachnospirales
Lachnospiraceae
Agathobacter
Roseburia
Oscillospirales
Ruminococcaceae
Faecalibacterium
Nodes
Change in median proportions
ASV count
-2.000
-0.889
-0.222
0.000
0.222
0.889
2.000
1.00
2.72
7.89
16.50
28.60
44.10
63.00
b) AD – Moist vs. Dry
Bacteria
Firmicutes
Bacilli
Bacillales
Staphylococcales
Staphylococcaceae
Staphylococcus
Bacillaceae
Bacillus
Lactobacillales
Streptococcaceae
Streptococcus
Peptostreptococcales-Tissierellales
Peptostreptococcales-Tissierellales_fa
Anaerococcus
Fenollaria
Clostridia
Lachnospirales
Lachnospiraceae
Blautia
Negativicutes
Veillonellales-Selenomonadales
Veillonellaceae
Veillonella
Proteobacteria
Alphaproteobacteria
Sphingomonadales
Sphingomonadaceae
Sphingomonas
Gammaproteobacteria
Salinisphaerales
Salinisphaeraceae
Salinisphaera
Pseudomonadales
Moraxellaceae
Acinetobacter
Burkholderiales
Oxalobacteraceae
Herbaspirillum
Actinobacteriota
Actinobacteria
Micrococcales
Micrococcaceae
Kocuria
Micrococcus
Microbacteriaceae
Curtobacterium
Leifsonia
Propionibacteriales
Propionibacteriaceae
Cutibacterium
Corynebacteriales
Corynebacteriaceae
Corynebacterium
Actinomycetales
Actinomycetaceae
Actinomyces
Change in median proportions
c) AD – Dermal vs. Epidermal
Bacteria
Actinobacteriota
Actinobacteria
Corynebacteriales
Corynebacteriaceae
Corynebacterium
Propionibacteriales
Propionibacteriaceae
Cutibacterium
Micrococcales
Micrococcaceae
Rothia
Kocuria
Coriobacteriia
Coriobacteriales
Coriobacteriaceae
Collinsella
Proteobacteria
Alphaproteobacteria
Rhizobiales
Rhizobiaceae
Brucella
Gammaproteobacteria
Xanthomonadales
Xanthomonadaceae
Vulcaniibacterium
Pasteurellales
Pasteurellaceae
Haemophilus
Firmicutes
Bacilli
Staphylococcales
Staphylococcaceae
Staphylococcus
Lactobacillales
Streptococcaceae
Streptococcus
Carnobacteriaceae
Carnobacterium
Clostridia
Peptostreptococcales-Tissierellales
Peptostreptococcales-Tissierellales_fa
Anaerococcus
Change in median proportions

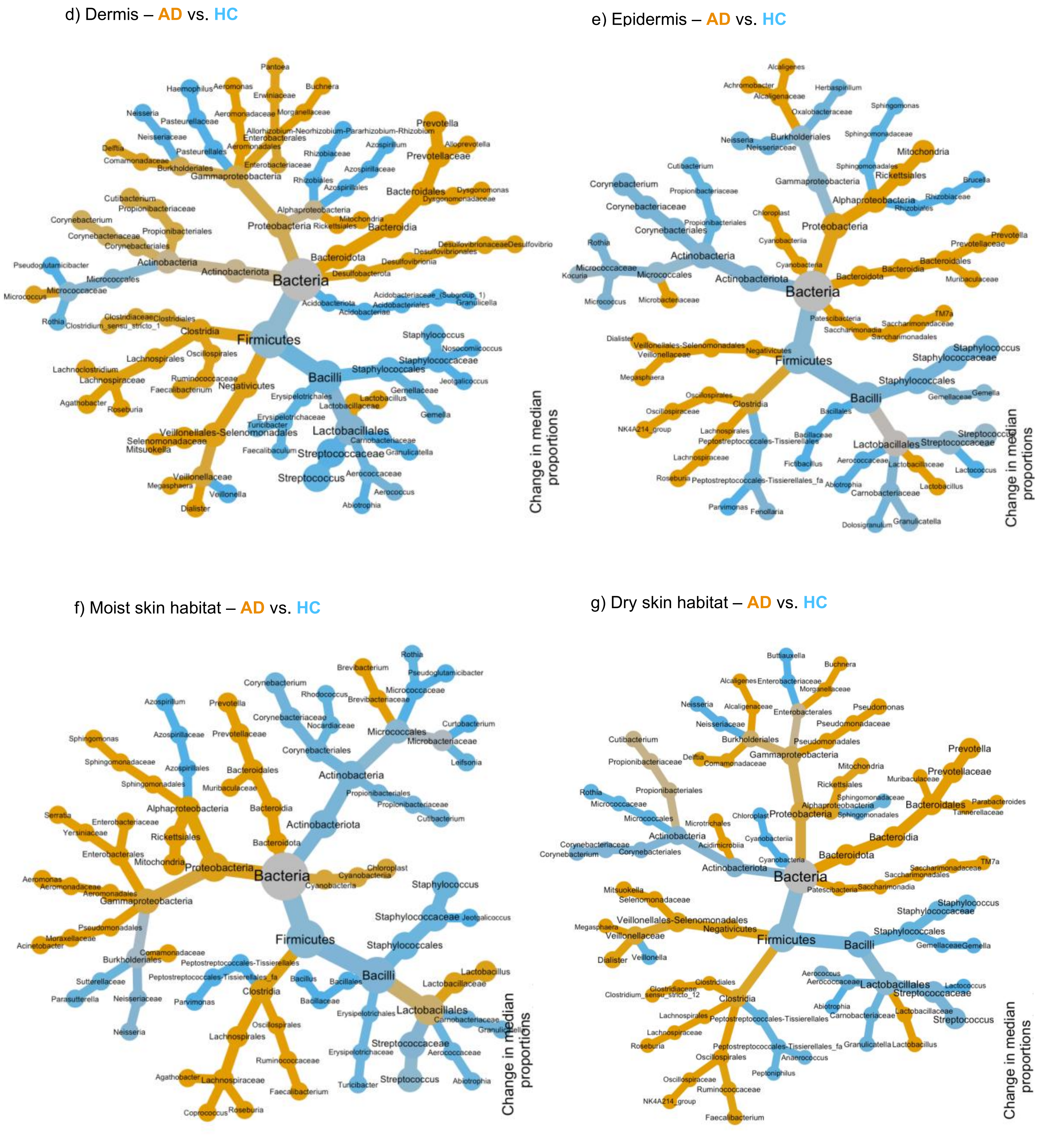

Supplementary Figure S4: Bacterial Specificity in AD and Healthy Skin. Heat trees of ASVs differing between the bacterial community within a) the entire skin of AD (orange) and healthy (blue), the AD bacterial communities of b) moist (blue) and dry (orange) skin habitats and c) epidermal (blue) and dermal (orange) skin compartments, the AD (orange) and healthy (blue) dermis d), and epidermis e) skin habitats, and the moist d) and dry e) skin habitats. Grey nodes are equally represented in both comparing groups.
