# Supplementary material for: Unique dermal bacterial signature differentiates atopic dermatitis skin from healthy
Source: mSphere. 2025 May 9;10(6):e00156-25. doi: 10.1128/msphere.00156-25 (PMC12188723; doi:10.1128/msphere.00156-25)
Supplement: Fig. S1 to S3 — Scattered, box, and bar plots of additional data. [file msphere.00156-25-s0002.pdf]

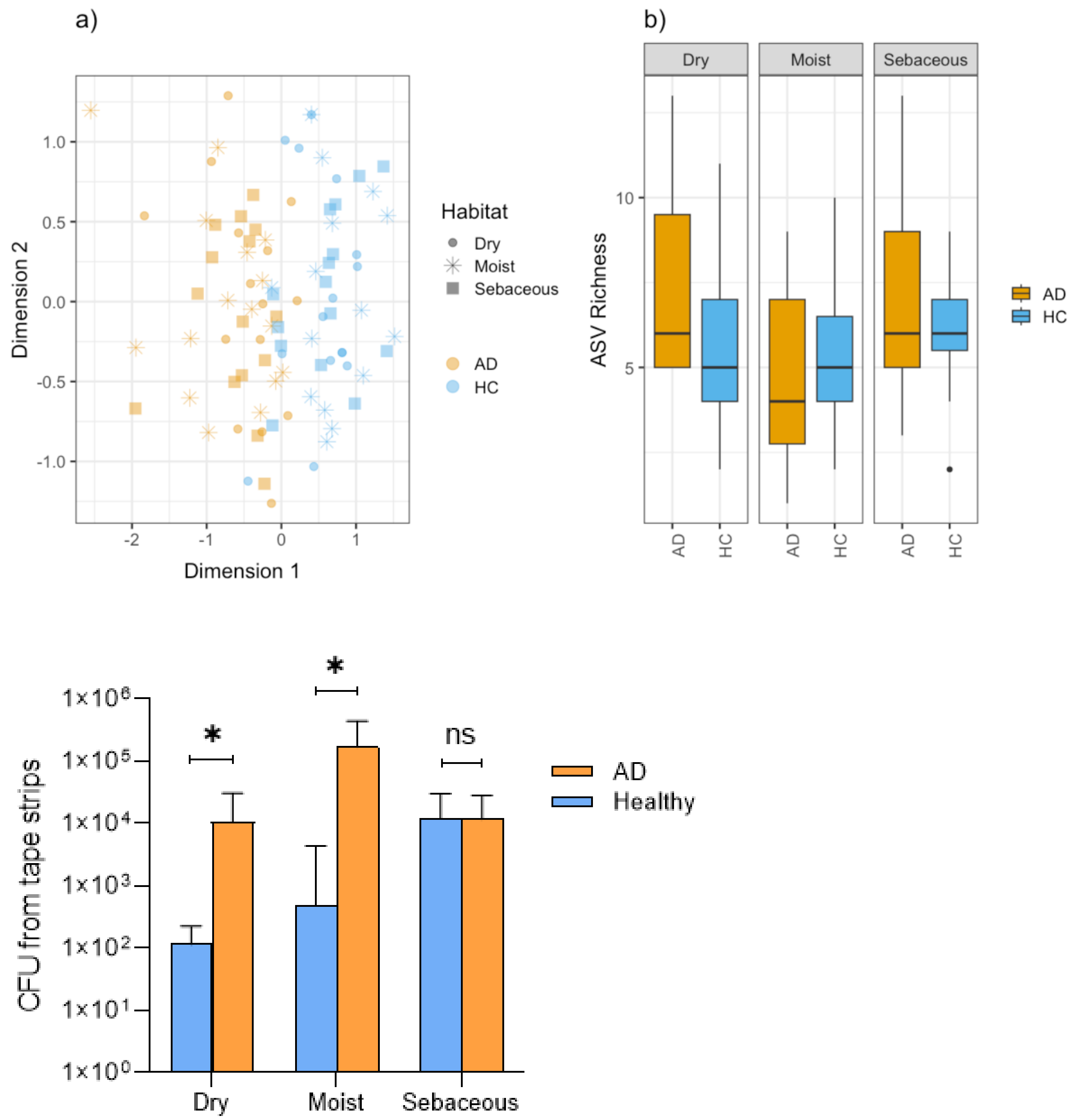


**Supplementary Figure S1:** a) Non-metric multidimensional scaling plot of the cultivated bacterial community facetted by their skin habitat, with colour representing AD (orange) and healthy controls (HC) (blue) and shape representing the skin habitats (circle = dry, star = moist, and square = sebaceous). b) Box plots visualising the ASV richness of the cultivated bacterial communities similarly divided by AD status and skin habitat. c) A bar chart of the viable bacteria (colony forming units [CFUs]) collected by tape strips in 15 layers at dry and moist (AD lesional and non-lesional) and sebaceous habitats (AD non-lesional) and corresponding healthy (HC) skin habitats and locations.

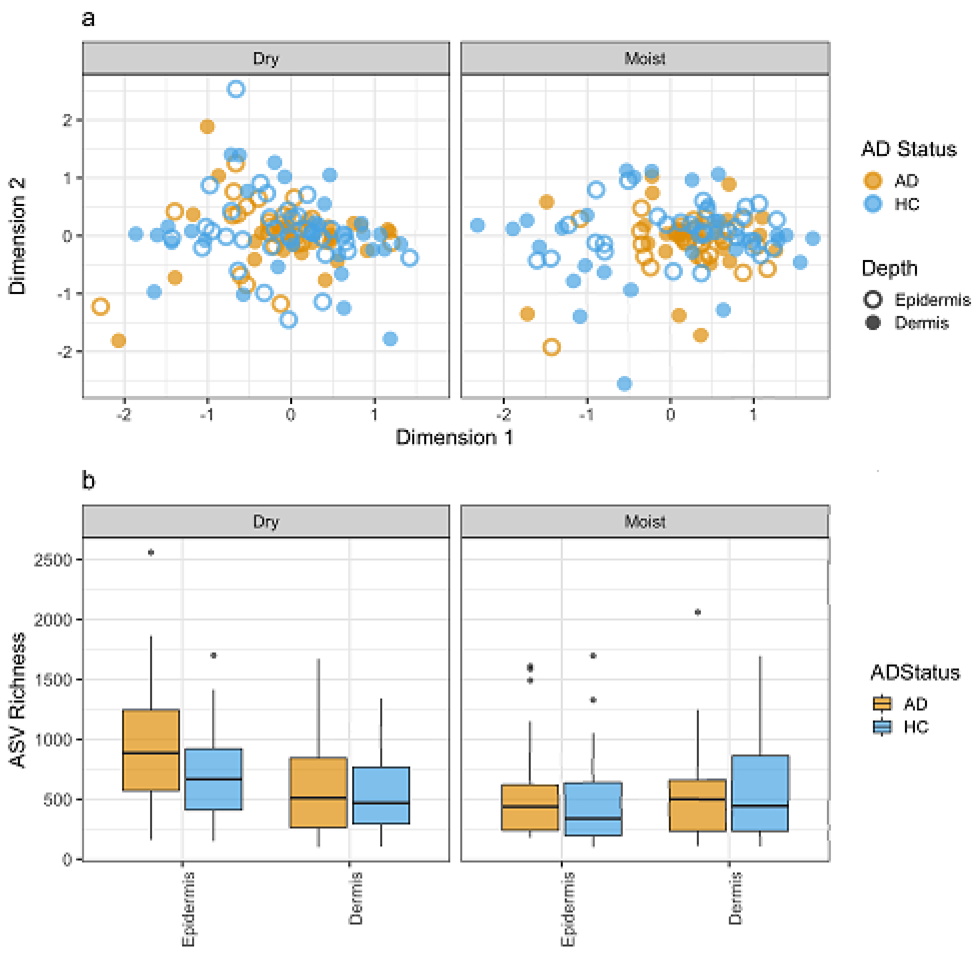


**Supplementary Figure S2:** a) Non-metric multidimensional scaling plot of the sequenced bacterial community, with colour representing atopic dermatitis (AD - orange) and healthy controls (HC - blue) and filling representing the skin compartment (ring=epidermis, filled circle= dermis). Samples were facetted by their skin habitat. b) Box plots visualising the ASV richness of the bacterial communities similarly divided by skin layer, AD status and habitat.

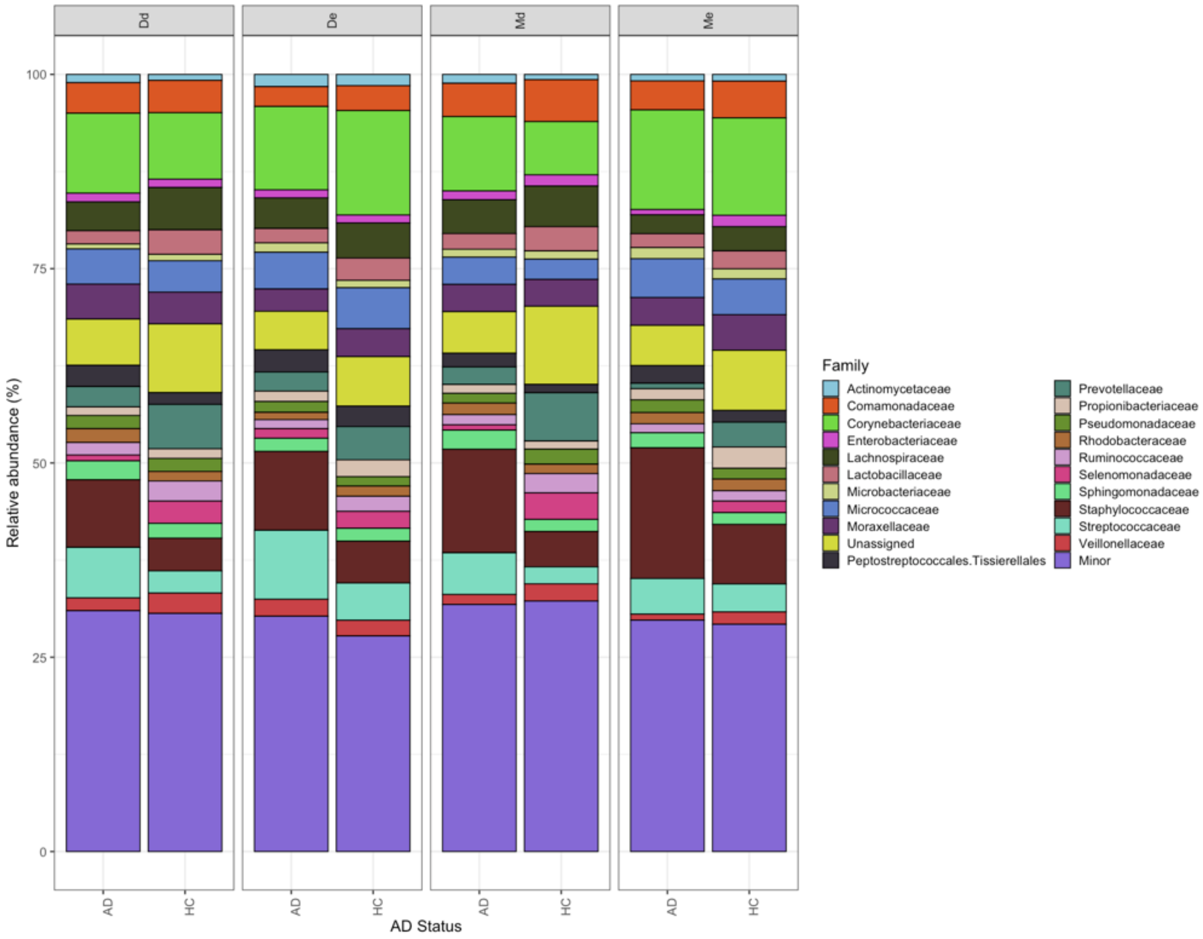


**Supplementary Figure S3: Bacterial Specificity in Skin Habitats in atopic dermatitis and Healthy Controls.** Stacked bar chart of the relative abundance (%) of bacterial families between atopic dermatitis (AD) and healthy controls (HC) within the four skin compartments: Dry dermis (Dd), Dry epidermis (De), Moist dermis (Md), Moist epidermis (Me).
